# Interactive and Participatory Audit and Feedback (IPAF): theory-based development and multi-site implementation outcomes with specialty clinic staff

**DOI:** 10.1186/s43058-021-00155-4

**Published:** 2021-05-31

**Authors:** Edmond Ramly, Diane R. Lauver, Andrea Gilmore-Bykovskyi, Christie M. Bartels

**Affiliations:** 1 Department of Family and Community Medicine, School of Medicine & Public Health, University of Wisconsin-Madison, Madison, WI USA; 2 Department of Industrial and Systems Engineering, College of Engineering, University of Wisconsin-Madison, Madison, WI USA; 3 School of Nursing, University of Wisconsin-Madison, Madison, WI USA; 4 Department of Medicine, School of Medicine & Public Health, University of Wisconsin-Madison, 1685 Highland Ave, Rm 4132, Madison, WI 53705-2281 USA

**Keywords:** Audit and feedback, Theory-based strategies, Implementation strategies, Implementation outcomes, Effectiveness-implementation hybrid, Frontline staff, Specialty clinics, Preventive care, Cardiovascular disease, Medical assistants

## Abstract

**Background:**

Theory-based implementation strategies, such as audit and feedback (A&F), can improve the adoption of evidence-based practices. However, few strategies have been developed and tested to meet the needs of specialty clinics. In particular, frontline staff can execute cardiovascular disease (CVD) risk reduction protocols, but A&F strategies to support them are not well examined. Our objective was to develop and evaluate a theory-based approach to A&F, Interactive and Participatory A&F (IPAF).

**Methods:**

We developed IPAF informed by two complementary theories, self-regulation theory (SRT) and self-determination theory (SDT). IPAF applies concepts from these theories to inform (1) what to address with staff to improve rates of best practices (SRT) and (2) how to interact with staff to improve behaviors aligned with best practices (SDT). We promoted IPAF fidelity by developing a semi-structured guide to facilitate staff discussion of target behaviors, perceived barriers, goals, and action plans. We evaluated IPAF in the context of eight quasi-experimental implementations in specialty clinics across two health systems. Following a hybrid type 2 effectiveness-implementation design, we reported intervention outcomes for CVD risk reduction elsewhere. This paper reports implementation outcomes associated with IPAF, focusing on feasibility, appropriateness, acceptability, fidelity, and adoption. We evaluated implementation outcomes using mixed-methods data including electronic health record (EHR) data, team records, and staff questionnaire responses.

**Results:**

Eighteen staff participated in 99 monthly, individual, synchronous (face-to-face or phone) IPAF sessions during the first 6 months of implementation. Subsequently, we provided over 375 monthly feedback emails. Feasibility data revealed high staff attendance (90–93%) and engagement in IPAF sessions. Staff highly rated questionnaire items about IPAF acceptability. Team records and staff responses demonstrated fidelity of IPAF delivery and receipt. Adoption of target behaviors increased significantly (all *P* values < 0.05), and adoption or behaviors were maintained for over 24 months.

**Conclusions:**

We developed and evaluated a theory-based approach to A&F with frontline staff in specialty clinics to improve the implementation of evidence-based interventions. The findings support feasibility, appropriateness, acceptability, and fidelity of IPAF, and staff adoption and maintenance of target behaviors. By evaluating multi-site implementation outcomes, we extended prior research on clinic protocols and A&F beyond primary care settings and providers.

**Supplementary Information:**

The online version contains supplementary material available at 10.1186/s43058-021-00155-4.

Contributions to the literature

- By developing and implementing new clinic protocols to control CVD risk factors using a theory-based audit and feedback (A&F) strategy with frontline specialty staff, we have built upon prior research on protocols from primary care and on A&F with providers.
- We offered supporting evidence to replicate and extend IPAF in future implementations by demonstrating sustained staff behavior changes across eight implementations of two distinct interventions addressing blood pressure follow-up and tobacco cessation referral.
- Our detailed report on our application of two complementary theories to develop and assess IPAF responds to experts’ calls to explicate rationale and applications of theory in implementation science.

## Background

Audit and feedback (A&F) is an established implementation strategy [1] to improve uptake of evidence-based practices, but its effectiveness remains variable across studies, with effect sizes often stagnating since 2003 [2, 3]. Implementation science experts argue that applying theory to design, measure, and report implementation strategies can strengthen implementations [2, 4–7]. Yet, in the most recent Cochrane review of 140 A&F trials to improve professional practice and patient outcomes, only 14% of studies (*n =* 20) used theory to inform A&F and only 9% (*n* = 13) reported the details of how the theory was applied [5]. In addition, key characteristics of how A&F strategies were designed and used are often missing or inconsistently addressed [4, 5]. Using theories can address key characteristics previously identified to explain the varied effect sizes in prior studies, including the format and frequency of feedback, the role of persons giving feedback, the type of interpersonal interaction, transparency of desired clinical outcomes toward which implementation efforts are addressed, and whether instructions are provided about specific future goals and action plans [4, 5]. Implementation science could benefit from explicit descriptions of theories applied to implementation strategies in general and to A&F, in particular, to improve efficacy and replicability by leveraging existing knowledge on behavior change. Important details include the rationale for theory selection, details of how concepts are applied, and explication of how key characteristics of A&F correspond to the theory. With greater clarity and specificity about the application of theoretical concepts to A&F, knowledge in the field can be built more efficiently, with the intent to increase effect sizes.

A&F can promote guideline-concordant care by improving how health care professionals follow clinical practice protocols but has primarily been studied with physicians, nurses, and pharmacists [5]. Evidence-based protocols can be executed by frontline staff such as medical assistants. This is well illustrated in the area of cardiovascular disease (CVD) prevention, where clinic protocols executed by medical assistants have improved the proportion of primary care clinic patients whose blood pressure (BP) is controlled from 50 to > 80% [8]. In specialty clinics, however, clinicians discuss CVD risk factors such as BP or tobacco in only 10% of relevant specialty visits, despite routinely assessing them [9, 10]. Non-vascular specialty clinics have not implemented protocols to address high BP and tobacco use, which are the most prevalent risk factors for CVD in US adults. In particular, although many rheumatology populations face increased inflammatory CVD risks [11], rheumatologists typically consider addressing CVD risk factors to be outside their scope of practice. Because frontline staff routinely assess risk factors such as BP and tobacco, integrating evidence-based protocols could promote routine action to address immediate care and follow-up needs. If specialty clinics employed CVD risk reduction protocols, then they could reach many more of the two million specialty patients who have CVD events annually, recognizing that specialty visits are nearly equal to primary care visits in the USA [12]. The target behaviors for our specialty clinic protocol to align with CVD guidelines are for specialty frontline staff to *Check* a CVD risk factor (e.g., blood pressure verification or smoking and readiness to quit), *Advise* the patient on the risk factor’s relation to their condition (e.g., rheumatoid arthritis), and *Connect* the patient to risk reduction resources (e.g., primary care or quit line) [13, 14]. We reported elsewhere the intervention outcomes of *Check, Advise*, and *Connect* clinic protocols for timely patient follow-up in primary care after high BPs and referrals to the tobacco quit line after assessing readiness to quit [13–16].

Our objective was to develop, operationalize, and evaluate a theory-based A&F strategy. Specifically, we aimed to:

1. Develop a theory-based A&F strategy based on behavioral and motivational theories
2. Operationalize the proposed strategy with a tool to guide A&F session fidelity
3. Evaluate the proposed A&F strategy in the context of eight quasi-experimental implementations of evidence-based interventions to reduce CVD risk

We named the proposed strategy Interactive and Participatory A&F (IPAF). We describe the proposed strategy as *interactive* because rather than only offering unidirectional feedback, the facilitator both requests feedback from the staff about their barriers to engaging in the target behaviors and offers feedback to staff on their recent behaviors. Then, the facilitator and staff discuss and agree on short-term goals and action plans to improve target behaviors to align with the intervention goals (e.g., blood pressure control, smoking cessation). We further describe the proposed strategy as *participatory* because the facilitator invites the staff to make their own choices: choosing whether to give or receive feedback first, setting their own short-term goals to focus on, and deciding their own action plans for overcoming their barriers and achieving their goals.

We operationalized the proposed strategy with a tool consisting of a semi-structured guide that the facilitator used during A&F sessions to complete all the steps of IPAF. The tool had six sections: introduction, clarifying the purpose of the meeting, offering choice, obtaining feedback from staff, sharing feedback with staff and setting goals, and planning action steps.

Our evaluation of IPAF focused on the implementation outcomes of feasibility, appropriateness, acceptability, fidelity, and adoption [17]. By reporting on the application of IPAF in eight implementations as an exemplar, this paper describes the methodology to enable others to provide theory-based, interactive, and participatory feedback to promote target behaviors that health professionals need to perform to align with evidence-based recommendations. In this evaluation, the target behaviors were for specialty frontline staff to check a CVD risk factor, advise the patient on the risk factor’s relation to their specialty condition, and connect the patient to risk reduction resources.

## Methods

### Theory-based development of IPAF

Consistent with calls to apply theory to the development of interventions and implementation strategies [5, 6, 18], we applied theory to develop a theory-based A&F strategy and guide relevant evaluation measures. We selected the self-regulation theory (SRT) and self-determination theory (SDT) [19, 20], which are middle-range theories and are readily applied in practice [21]. Our choice of SRT was guided by evidence from, and recommendations in, a Cochrane review of A&F, and SDT has received empirical support from prior research with adult participants in a variety of worksites [19, 21]. SRT concepts guided us in identifying what components to address for behavior change (e.g., referent points for intervention outcomes). However, SRT concepts were not sufficient to inform us on how to create social environments in which staff would be likely to adopt. Because SDT can inform how to interact with staff to improve their behaviors aligned with new initiatives, we chose to apply SDT concepts also. Both theories offer clarity, utility, and parsimony for behavior change with adults [19, 20]. The following two sections describe how SRT and SDT theories guided our A&F development and evaluation in a complementary manner.

#### How self-regulation theory guided IPAF development

We applied SRT to the development of IPAF as an implementation strategy, consistent with recommendations by experts in SRT [20, 22] and A&F [4, 5]. SRT involves a process of how people regulate their behaviors toward an adopted future state or goal. The concept of an ideal state (e.g., behavior) functions as a referent point for those involved. Importantly, SRT includes a hierarchy of corresponding or aligned goals from more abstract to more specific [20, 22]. Organizational leaders and individual staff can compare current rates of behaviors against the same goal (e.g., to have 60% of the patient population vaccinated). The consequences of engaging in comparisons between real and ideal states result in conclusions of either congruence or discrepancy between states. Discrepancy typically is motivational for people to regulate their own behaviors so that they may observe more congruence alignment between their future behaviors and ideal states [6, 20, 22].

**Fig. 1:**
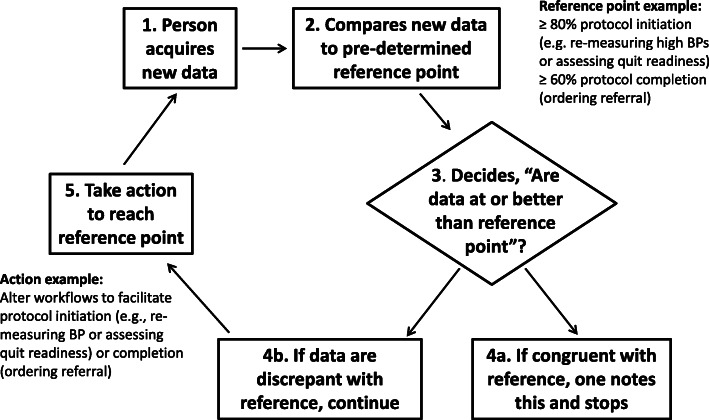
Steps based on the concepts in self-regulation theory and applied to IPAF in the context of CVD risk reduction. Starting at step 1 (top left), a person acquires new data. At step 2, compares new data to a pre-determined and desired reference point. Then decides (3) whether or not data are congruent with the reference point. If yes, record and stop (4a). If discrepant with the reference point (4b), take action (5) for improvement so future data would be congruent with the reference point. These steps apply for clinic staff comparing their performance to desired performance goals or patients targeting health goals

As an illustration, assume an intervention goal is to increase rates of BP re-measurements to 80% (an ideal) among patients whose initial BPs were high. This goal provides a reference point or standard against which clinicians can compare staff’s rates of repeat BPs in an audit (Fig. 1). In the context of our applications, if staff receive feedback that their behaviors are inconsistent with intervention goals that they agreed to support, then they likely would experience discrepancy. As a result, they would be motivated to improve their target behaviors, have future behaviors align with intervention goals, and experience less discrepancy in the future.

Consistent with SRT, specific behavior change techniques have been useful for adopting new behaviors. Techniques include barrier identification, goal setting, and action planning [23], as shown in Table 1. These techniques have been associated with desired behavior change across different types of behaviors [23, 24] and proposed for application in A&F in particular [3, 5]. These behavior change techniques are relevant to our staff’s processes of monitoring and adopting target behaviors.

**Table 1:** Theoretical rationale for elements of IPAF and corresponding guide

Elements addressed in delivery of IPAF		Applications of Concepts from Theory*	Theory
**I. Introduction**			
A	Show respect for staff, creating positive interpersonal environment.	Building relatedness, interacting in a humanistic manner	SDT
B	Share that facilitator will use a written guide for consistency.	Knowing what to expect can give staff realistic expectations and reduce uncertainty	SRT
**II. Clarifying purpose of meeting**			
A	Clarify overall purpose of meeting with IPAF.	Being transparent can give staff clarity of expectations for their behaviors.	SRT
B	Specify purpose is not to judge staff’s job performance.	Reduce anxiety, improve attention; Non-judgement interaction can support staff participation.	SRT, SDT
C	Share topics for meeting; To gain feedback from staff about their experience implementing target behaviors and to provide staff feedback on target behaviors.	Treating staff as colleague facilitates relatedness and perceived competence and can support corresponding staff behavior.	
D	Review altruistic purpose (e.g., screening & referral for CVD risk), the intervention goals	Sharing rationale can assist staff to endorse long-term goal of study to which they can align their specific goals and behaviors.	SRT
E	Facilitator invites discussion of contextual factors that influence target behaviors and Invites exchange of information.	Staff likely to believe leaders understand their situation, supporting motivation for behavior.	SDT
**III. Offering choice**			
A	Offer meaningful choices re: what to discuss first: staff feedback to facilitator or facilitator’s feedback to staff.	Maintaining relatedness, respect and autonomy can support staff participation.	SDT
**IV. Obtaining feedback from staff**			
A	Request feedback from staff on facilitators to engaging in target behaviors.	Focus on staff capability and desirable behavior can facilitate relatedness and perceived competence; Focus staff’s attention on their recent behaviors in comparison to intended outcomes; Elicits participation.	SRT SDT
B	Request feedback from staff on barriers to engaging in target behaviors. *(barriers identification)*	As above. Avoids undermining staff perceived competence. Recognition of contextual influences on behaviors can improve staff motivation for target behaviors.	SRT SDT
**V. Sharing feedback on performance data with staff**			
A	Focus staff’s attention on their recent behaviors.	Can stimulate comparison of current behavior vs. reference point; Can result in discrepancy or congruence, which can stimulate motivation to improve or maintain behavior.	SRT
B	Offer choices for staff setting their short-term behavioral goals. *(goal setting)*	Facilitating participation. Supporting autonomy. Directing attention to short-term goals and intervention outcomes can be motivating.	SRT SDT
**VII. Planning action steps**			
A	Offering choice of action plans, setting up situation for building perceived competence to improve target behaviors and intervention goals. *(action planning)*	Facilitating participation. Supporting autonomy, perceived competence, and motivation for adoption of target behaviors.	SDT SRT

*Concept descriptions and assumptions are clarified on first mention. For brevity, subsequent mentions state only the concept. *Abbreviations*: *SRT* Self-Regulation Theory, *SDT* Self-Determination Theory

We also applied SRT broadly to the interventions and overall implementation approach, as shown in Fig. 1. Using SRT, researchers have described, explained, and predicted how people manage (i.e., regulate) themselves to reach their goals over time. At the overarching level of the clinic protocol, SRT applies to our choosing reference points to control CVD among the patient population, that is, high BP or readiness to quit tobacco. At the staff and patient levels, SRT applies to individuals; people need clear reference points against which to evaluate health risk factors and decide whether they are present or not. In summary, SRT involves *what* to address to improve behavior.

#### How self-determination theory guided IPAF development

We applied SDT, a theory regarding motivation and behavior [19, 25, 26], to guide the development and evaluation of IPAF, as shown in Table 1. According to SDT, all people have three inherent psychological needs relevant to behavior: relatedness, autonomy (i.e., choice), and perceived competence (i.e., self-efficacy) [19, 26]. When these needs are met, people are more motivated to engage in relevant behaviors [19, 26]. Randomized, controlled studies, based on SDT, have demonstrated improvements in work behaviors [26–28]. If the staff’s psychological needs were met, then the staff would be more motivated to adopt target behaviors than if their psychological needs were ignored or thwarted. An underlying proposition is that the staff are more likely to be motivated to align their behaviors toward altruistic goals (e.g., intervention goals of reducing CVD risk) when they experience autonomous rather than controlled environments. Accordingly, we designed IPAF to meet the staff’s psychological needs during individual feedback sessions. In summary, SDT involved how to interact with the staff to achieve both their target behaviors and intervention goals.

### Application of IPAF as an implementation strategy

#### Design

We evaluated implementation outcomes associated with IPAF in the context of a broader pre-, post-, quasi-experimental evaluation of CVD risk reduction interventions [13]. That evaluation followed a hybrid type 2 effectiveness-implementation design [29], attending to both intervention and implementation outcomes. We reported elsewhere the intervention outcomes: (a) timely patient follow-up in primary care after high BPs and (b) referrals to the tobacco quit line after assessing readiness to quit [13–16]. In this paper, we report on evaluations of the implementation outcomes of feasibility, appropriateness, acceptability, fidelity, and adoption [17] focusing on measures relevant to IPAF. We delivered IPAF sessions to individuals synchronously (in-person or by phone) for 6 months after the beginning of implementation and, later, asynchronously by email for over 24 months. Table 2 shows the components of the CVD risk reduction interventions and the implementation package within which IPAF was used to provide feedback.

**Table 2:** Intervention and implementation components: context of IPAF application

**Intervention components (target behaviors)**	
1) *Check* risk factor	
2) *Advise* patient on risk factor relation to specialty condition	
3) *Connect* patient to risk reduction resources	
**Implementation components (strategies)**	
1) *Engage* staff in planning implementation	
2) *Educate* staff about protocols and rationale	
3) *Remind* staff of protocol steps using electronic health record alerts	
4) *Feedback* based on audits of staff’s target behaviors, with ***IPAF approach***: interactive, participatory discussions about barriers, solutions, goals, and in which staff chose their own action plans to improve their behaviors to align with intervention goals	

#### Setting and sample

We evaluated IPAF in eight implementations, representing two interventions (BP Connect and Quit Connect) [13, 16, 30–32] in four separate rheumatology clinics in two US health systems. Clinics A, B, and C were in a large, suburban, academic, multi-specialty practice; clinic D was a community clinic. Rheumatology clinics offer an ideal setting and specialty population to evaluate A&F with frontline staff as a strategy to implement CVD risk reduction [8, 33, 34]. Our IPAF participants were all medical assistants and nurses who performed pre-visit rooming (i.e., vital signs, patient history) at the clinics. We collected mixed-methods data including their responses to questionnaires, EHR data, and team records.

#### Context

##### Intervention components

The components of our interventions and implementation package are shown in Table 2. We sought to improve the staff’s target behaviors with two CVD risk reduction interventions: BP Connect for high BP [13, 30] and Quit Connect for tobacco use [16, 31]. With a *Check*-*Advise*-*Connect* structure for both interventions, the target behaviors were to *check* for addressable risk factors, confirming high BPs or readiness to quit tobacco; *advise* patients on CVD risk; and *connect* patients to relevant resources. Connecting consisted of offering follow-up arrangements for BP appointments with primary care or for quit line phone calls for tobacco cessation counseling.

##### Implementation components

**Fig. 2:**
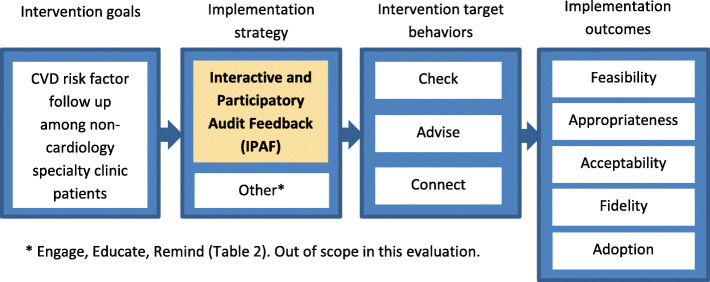
Logic model of the evaluation of IPAF as an implementation strategy

Our multi-faceted implementation package consisted of four implementation components as described in Table 2: Engage, Educate, Remind, and Feedback. This paper focuses on the Feedback component, which is the application of IPAF that we describe in detail below (Fig. 2). We engaged the staff in tailoring focus groups and reminded the staff using EHR alerts to check/advise and connect via EHR referral orders as detailed in our toolkits https://www.hipxchange.org/AuditFeedback [35]. For the Educate component, we held 1-h educational sessions with the staff in small groups at the beginning of each implementation. We explained the rationale, principles, and components for the intervention and implementation. We shared relevant evidence to address BP and tobacco, encouraged interactive discussion, and provided scenarios for staff role-plays regarding the *Check-Advise-Connect* behaviors. These staff had not previously had responsibilities for confirming or addressing CVD risk factors with patients (i.e., BP level or readiness to quit tobacco) or for referring patients to resources (i.e., primary care or quit line). The interactive educational sessions concluded with staff demonstrating mastery of role-play dialog and navigation of the EHR (from the Remind component) and receiving information about the monthly, individual feedback they would receive, according to IPAF.

#### Interactive and Participatory Audit and Feedback

IPAF sessions consisted of three theory-based components:

1. Providing feedback to individual staff about their actual rates of target behaviors, directing their attention to ideal target behaviors which would align with the intervention, and stimulating comparisons between their behavioral rate and the ideal, resulting in discrepancy or congruence based on SRT
2. Interactive, one-on-one discussions of the staff’s experiences, including barriers and goals for target behaviors, while simultaneously supporting the staff’s psychological needs, based on SDT and SRT
3. Eliciting action plans with the staff about how they could improve rates of target behaviors, based on SRT, SDT, and evidence for behavior change techniques [23]

Consistent with best practices from the most recent Cochrane review of A&F [5], we delivered feedback monthly, individually, and face-to-face when possible, by a respected colleague (not a supervisor), to improve the staff’s target behaviors. The *source of feedback* (i.e., A&F facilitator) in clinics A, B, and C was a physician known to the staff, a leader in the settings, not a direct supervisor of the staff, and the project’s principal investigator (CB). In clinic D, the facilitator was a nurse researcher from another organization, with expertise in supporting nurses, known to the staff only from engagement activities (AGB). The *context for feedback* with individual staff was synchronous for the first 6 months of implementation, in-person for clinics A, B, and C and by phone for clinic D. The IPAF facilitator met with individuals for up to 10 min, privately in a clinic room or by phone, at a mutually agreed time. After the first 6 months of each implementation, we shared feedback asynchronously by email, along with questions for the staff to share their barriers, goals, and action steps regarding target behaviors. The staff sent their responses and goals to the facilitator by email. The *frequency of feedback* was monthly amounting to at least four synchronous sessions per individual between months 1 and 6 of each implementation, and over 375 monthly emails for up to 4 years thereafter (2016–2019).

**Fig. 3:**
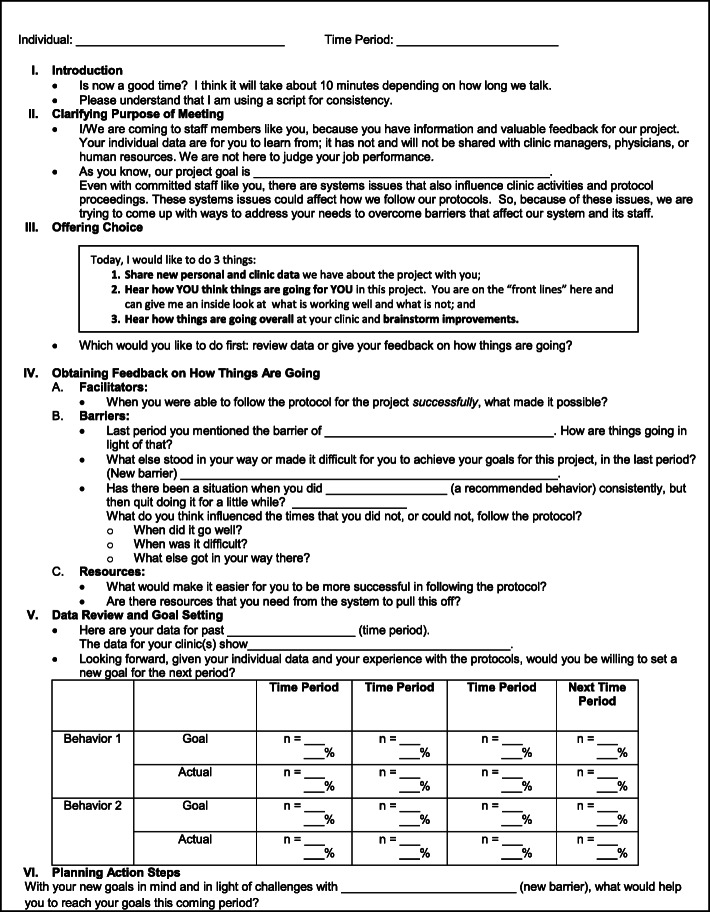
Tool to guide the application of Interactive and Participatory Audit and Feedback (IPAF) with fidelity

Facilitators and participants collaboratively used the IPAF tool during the synchronous IPAF sessions, shown in Fig. 3. The purpose of the tool was to serve as a guide, to support fidelity of delivery and receipt [17, 36]. Rather than being a rigid script, the tool consisted of a worksheet that was semi-structured to guide flexible and realistic discussions. It included *what* critical components to address with the staff to improve clinic rates of best practices (self-regulation theory) and *how* to interact with the staff to improve their behaviors aligned with best practices (self-determination theory) based on SDT. Table 1 presents the concepts, theories, and rationale for the IPAF components included in the tool.

Feedback sessions addressed the SRT concepts depicted in Fig. 1. Guided by the IPAF tool (Fig. 3), the facilitator provided feedback on individuals’ rates of target behaviors and shared the range of peers’ rates of target behaviors, grouped by clinic. Feedback was designed to stimulate the recipients’ own comparison between their recent behaviors and individual target behaviors, as well as with intervention goals. Also, the IPAF facilitator explained the long-term goal of controlling CVD in the rheumatology population to highlight an altruistic and non-controlling motive to improve one’s behaviors. When the staff experienced a potential discrepancy between actual and desired behaviors, then we assumed this could be motivational for new goal setting and regulation.

Feedback sessions also addressed SDT concepts [25]. Guided by the IPAF tool (Fig. 3), the IPAF facilitator explained that the intent was to be collaborative, not judgmental. To respect individuals’ autonomy, the facilitator offered choices about the order of discussion topics, starting with either feedback on individual-level data or discussing how the individuals thought the intervention was going. The facilitator elicited from individuals their barriers to engaging in target behaviors, possible solutions, goals for target behaviors, and action steps for the upcoming month.

### Evaluation of implementation outcomes associated with IPAF

#### Data collection

##### Feasibility

We evaluated feasibility by whether we were able to collect rates of individual staff’s target behaviors. The staff were to document these behaviors in new EHR data fields to enable monthly audits. The information technology staff were to report rates of these behaviors monthly to the implementation team. Additionally, we evaluated the degree of staff participation in IPAF sessions, based on team records including notes from facilitators and debriefing session facilitators had with other team members after each IPAF session. We assessed both staff’s attendance at feedback sessions and their engagement in planned steps for behavior change during these sessions.

##### Appropriateness

We assessed appropriateness qualitatively during meetings to engage the staff in planning (Engage implementation component) pre-implementation and at 3 months mid-implementation.

##### Acceptability

We evaluated the staff’s opinions with selected items from a 22-item written questionnaire. Questionnaires developed by the study team included items to evaluate acceptability and fidelity to theory components [19, 20, 22, 25, 26] and support for the work system [37, 38]. For example, consistent with SDT concepts, participants reported the degree to which the project team listened to them, responded to their ideas, flexibly tailored protocols, addressed barriers, and collaborated in planning. The staff answered anonymously at month 6 on a 5-point scale from 1 “not at all” to 5 “extremely”. We also asked the staff for comments at the end of the questionnaires. Finally, team records, including facilitator notes and post-session debriefings, documented the degree of participants’ engagement during the one-to-one feedback sessions.

##### Fidelity

We evaluated fidelity based on monthly copies of the IPAF tool and on staff questionnaire responses. Completion of all sections of the IPAF tool by the facilitator for each session indicated fidelity of delivery. We evaluated the fidelity of receipt with relevant questionnaire items, including feedback based on SDT and SRT. These included the extent to which participants felt respected by the staff, found problem solving helpful, and experienced goal setting as motivational during the feedback sessions.

##### Adoption

We measured staff’s rates of target behaviors, consistent with the *Check-Advise-Connect* structure and SDT/SRT reinforced A&F for the two interventions. We defined adoption of the *Check* behavior as the rate of confirming the CVD risk factor: re-measuring high BPs or asking about readiness to quit tobacco. We defined adoption of the *Connect* behavior as the rate of offering follow-up: appointments with primary care for high BP or electronic referrals for counseling calls from the tobacco quit line. Moreover, in addition to actual rates, our theories suggest that intentions and perceived confidence can predict behavior. On the monthly IPAF worksheets, we evaluated the staff’s goals for future rates of target behaviors to reflect intentions at the first and last interactive sessions. In questionnaires, we asked the staff to rate their perceived confidence in addressing CVD risk factors pre- and post-intervention, on a 5-point scale, retrospectively for clinics A, B, and C and prospectively for clinic D.

##### Analyses

We generated descriptive statistics, including frequencies and standard deviations, for responses to questionnaire items and target behaviors. Paired *t* tests were used to compare the staff’s pre- and post-questionnaire responses.

##### Protection of human participants

Our project received approval through the University of Wisconsin-Madison’s Health Sciences Institutional Review Board and the Gundersen Health System’s Institutional Review Board. According to the policies covering research activities at both institutions, our project met the exemption criteria for operational improvement activities, with permission to publish. We summarized participants’ data in aggregate and did not share individual audits with the supervisors of participants.

## Results

Across our eight implementations, 18 different staff participated in IPAF sessions, and we received 30 responses from them on the month 6 questionnaires. We had 100% response rates among participants employed at the time the questionnaires were administered. All but one staff (94%) were female and most had no prior research or quality improvement experience. Table 3 shows the characteristics of the IPAF participants and questionnaire respondents.

**Table 3:** Characteristics of participants

**Audit and feedback** (*n =* 18 staff)^a^	%
Registered nurse	50
Medical assistant	50
Female	94
**Questionnaires** (*n =* 30 staff)	%
Prior experience with quality improvement	40
Years of clinical experience	
<1	14
1–4	36
5–10	14
>10	36

^a^*n=*18 unique individuals (14 positions) over four 6-month periods

### Feasibility

Both the audit and feedback components of IPAF demonstrated feasibility. The information technology staff were able to retrieve all monthly rates of target behaviors from the EHR, including new EHR fields documented by the staff. We were able to provide feedback in 99 total monthly in-person or phone sessions with 18 different staff and over 375 monthly emails thereafter. Staff attendance at the feedback sessions was high: 90% at 72 of 80 planned sessions in clinics A, B, and C and 93% at 25 of 27 planned sessions in clinic D. When participants missed a session, they were absent from work for illness or personal reasons. The sessions lasted 7 min on average (range 3–10 min).

According to team records, including facilitator notes and completed copies of the IPAF tool, all participants engaged in identifying barriers to target behaviors, setting goals, and making action plans. In notes and debriefings, IPAF facilitators observed that the staff were willing and able to set goals when prompted. A review of the copies of the IPAF tool showed that when the staff were unable to set a percentage goal for infrequent behaviors, the IPAF tool accommodated setting a numerical goal (e.g., *n* = 2 quit line referrals). Across team records, a common approach was to mutually determine suggested goals based on prior rates, achievable expectations, and the facilitator’s sense of an individual’s motivation in the project overall.

### Appropriateness

The staff that we engaged in planning meetings pre- and mid-implementation qualitatively described IPAF as appropriate. They perceived it as fitting well with their workflows and described it as practicable and useful as part of the implementation plans.

### Acceptability

Responses to questionnaire items relevant to IPAF revealed high acceptability. In clinics A, B, and C, respondents (*n =* 20) reported that the project team listened (mean = 4.47 ± 0.76 standard deviation), were responsive (4.46 ± 0.73), flexible to ideas (4.22 ± 0.97), and collaborative (4.44 ± 0.53). Table 4 shows further details from all implementations. In open-ended questionnaire responses, the staff reported appreciating seeing their data and practicing what they would say with patients in both training and individual IPAF sessions. Several staff reported eagerness to disseminate the protocols and implementation strategies, including IPAF, to other specialty clinics.

**Table 4:** Data on implementation outcomes: acceptability, fidelity, and adoption

	BP Connect at Clinics A, B, C	Quit Connect at Clinics A, B, C	BP Connect at Clinic D^**a**^	Quit Connect at Clinic D^**a**^
	Mean (SD) unless otherwise indicated			
^**b**^**Acceptability** (questionnaire)	***n =*** **10**	***n =*** **10**	***n =*** **4**	***n =*** **6**
Listening	4.33 (1.00)	4.60 (0.52)	3.75 (0.96)	3.50 (1.38)
Responsiveness	4.22 (0.97)	4.70 (0.48)	3.75 (0.96)	4.00 (0.63)
Flexibility	4.22 (0.97)	NA	3.50 (1.29)	NA
Collaboration	4.44 (0.53)	NA	3.50 (1.00)	NA
^**b**^**Fidelity** (questionnaire)	***n =*** **10**	***n =*** **10**	***n =*** **4**	***n =*** **6**
Respectfulness	4.90 (0.32)	4.89 (0.33)	4.00 (0)	4.67 (0.58)
Problem solving	4.90 (0.42)	4.78 (0.44)	3.33 (0.58)	4.67 (0.58)
Motivation from goals	3.70 (0.82)	4.56 (0.73)	2.50 (1.29)	NA
Confidence (pre)	2.60 (1.07)	2.10 (1.10)	3.33 (0.81)	2.5 (0.55)
Confidence (post)	4.00 (0.47)	4.30 (0.67)	3.75 (0.50)	3.67 (0.52)
^**c**^**Adoption** (IPAF tool/ EHR data)				
	***n =*** **40**	***n =*** **32**	***n =*** **12**	***n =*** **15**
*Check* goals at first session	50-90%	75%-100%	NA	100%
*Check* goals at last session	75-95%	75%-100%	NA	70%-100%
*Check* actual at baseline	2%	3%	0%	90%
*Check* actual 6 months post	98%	100%	80% peak	98%
*Connect* goals at first session	50-100%	50%-95%	NA	33%-80%
*Connect* goals at last session	50-100%	80-100%	NA	80%-85%
*Connect* actual at baseline	0%	<1%	0%	<1%
*Connect* actual 6 months post	73%	76%	68%	53% peak

^a^BP Connect was implemented after Quit Connect in Clinic D and A&F sessions were by phone; ^b^Pre-post confidence was measured retrospectively for clinics A, B, C and prospectively for D; ^*c*^*Connect* step: offer primary care BP follow-up or tobacco quit line phone call; *Abbreviations*: *NA* data not available

### Fidelity

**Fig. 4:**
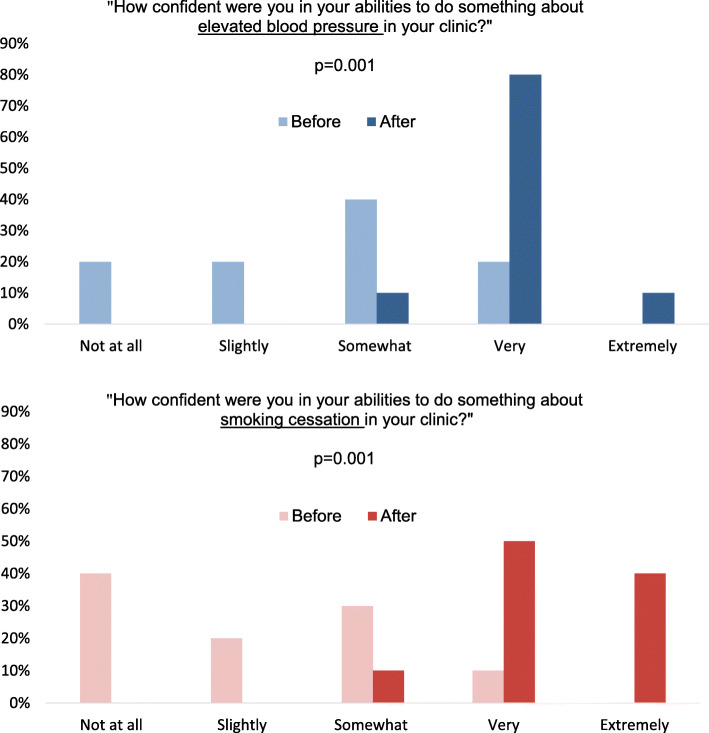
Staff scores on perceived confidence to address high BP and tobacco before and after implementation. Results show that 90% were very or extremely confident after (dark bar) compared to 10–20% before (light bar) each intervention (*n =* 10 respondents per questionnaire)

Data from copies of the IPAF tool documented the completion of >80% of all goal setting topics and other discussion sections, supporting fidelity of delivery. Detailed findings are in Table 4. To summarize, in clinics A, B, and C, respondents (*n =* 20) reported the following: the implementation team was respectful (4.90 ± 0.33), problem solving was helpful (4.79 ± 0.43), and goal setting was motivational (4.13 ± 0.78). When asked about perceived confidence addressing BP and tobacco, only 10–20% said they had been “very” or “extremely confident” at baseline while 90% reported they were “very” or “extremely confident” after 6 months (*P =* 0.001), as shown in Fig. 4.

Table 4 shows further details from all eight implementations including our replication site, clinic D. Overall, responses from implementations in clinic D showed lower scores. That health system was understaffed and had 66% clinic staff turnover during implementation, thus new staff received abridged engagement and education.

#### Addressing barriers

Using the IPAF tool to guide fidelity, facilitators asked participants to identify barriers to the adoption of target behaviors when prompted. These barriers included having high patient volume, being understaffed, working with float staff, and working with some providers who rushed processes for rooming patients. Participants also generated solutions for barriers during IPAF sessions. For example, participants needed to address a barrier of time pressure to measure and re-measure BPs before visits with providers. Participants offered a solution using red laminated reminder cards on the desks in patients’ exam rooms to alert providers and staff that repeat BPs were needed after provider visits. Using their solution resulted in a subsequent increase in rates of BP re-measurement.

#### Building rapport

In addition to the structured discussion, the monthly IPAF sessions guided by the semi-structured IPAF tool also afforded opportunities for reciprocal flexible communication, consistent with SDT. It was not uncommon for participants to share unsolicited details on changes in the work system or staff perceptions of the intervention or implementation process. Based on facilitator debriefings and participant comments, by having a consistent point of contact, facilitators also formed relationships with participants, so additional communication was able to occur. This fostered rapport and made going through structured feedback items more comfortable for both facilitators and participants.

### Adoption

As expected, adoption of target behaviors improved with monthly SRT- and SDT-based feedback. In the first IPAF sessions, the staff’s goals for the *Check* behavior varied from 50 to 100%. In contrast, in the last IPAF sessions, the staff’s goals for this behavior increased to 75–100%. Based on EHR data, the staff’s actual rates of the *Check* behavior improved from 2–3% to 98–100% pre- to post-implementation in clinics A, B, and C. Clinic D had a peak of 80% for BP Connect, though ranges varied while experiencing understaffing. The staff’s rates of the *Connect* behavior rose from 0–1% to 68–76% pre- to post-implementation, with the exception of Quit Connect at clinic D. Table 4 shows further details from all eight implementations.

## Discussion

### Advancing research on theory-based A&F as an implementation strategy

We have developed and evaluated a theory-based A&F strategy by applying a relatively novel combination of behavioral and motivational theories. Responding to the need for detailed reports of how theory guides A&F [4, 5, 39–42], we delineated the theoretical concepts informing our IPAF components and tool in Table 1 and Fig. 3, respectively. We have posted these resources as a toolkit at https://www.hipxchange.org/AuditFeedback [35]. SRT guided us in conceptualizing *what* to address, and SDT guided us in *how* to interact with the staff effectively. We applied concepts and evidence from SRT, supported by behavior change techniques [20, 22], which we incorporated into IPAF components, such as barrier identification and problem solving [20, 22, 23]. We applied SDT concepts to interpersonal processes during IPAF sessions to support the staff’s psychological needs and motivation for the adoption of target behaviors [26].

IPAF can be situated within the literature on behavior change through the Theoretical Domains Framework (TDF) which identifies influences on health professional behavior regarding implementation of evidence-based recommendations [43]. The TDF has classified SRT and SDT as action and motivation theories, respectively [44]. IPAF is consistent with most of the theoretical domains of the TDF. Specifically, our combination of SRT and SDT addresses 8 of 14 domains directly, three domains indirectly, and does not address three domains (optimism, emotion, intention). The strength of our approach is the parsimonious use of two middle-range theories as this combination provided concepts that were applicable and allows for testable relationships between the concepts to be examined in future research. We are also not aware of other detailed evaluations of implementation outcomes associated with theory-based or theory-informed A&F interventions.

More recently, Clinical Performance Feedback Intervention Theory (CP-FIT) [45] is an overarching theory of A&F, published after we completed the development and evaluation work reported here. The CP-FIT feedback cycle is consistent with IPAF, with an important distinction. CP-FIT conceptualizes A&F participants as recipients, which risks perpetuating a view of A&F as a passive, unidirectional delivery of information. IPAF demonstrates the benefits of an expanded view whereby A&F is a collaborative effort between facilitators and participants interacting to exchange feedback from participants about perceived barriers to target behaviors and from facilitators’ performance data and shared strategies, where participants can autonomously make choices about their goals and action plans. Our IPAF approach also adds to the Calgary Audit and Feedback Framework [46, 47] and the Relationship, Reactions, Content, Coach model (R2C2) [48]. Interactive and participatory processes may be particularly suited to A&F with health professionals with typically lower autonomy and perceived competence, including medical assistants versus physician-focused A&F approaches. The Calgary and R2C2 approaches were developed with a physician focus and use group A&F as a central component. Group A&F likewise requires further scrutiny in the context of frontline staff such as medical assistants who may feel intimidated in that setting, especially in groups including individuals with varying experience, tenure, and stature. The present IPAF study examined individual A&F, though we have begun comparing group vs. individual feedback with medical assistants in new studies.

### Advancing research on implementing clinic protocols with A&F

We have extended prior research on clinic protocols in primary care [8] and on A&F with physicians, pharmacists, and nurses by primarily targeting frontline medical assistants [5, 6, 8]. We found that IPAF is feasible, appropriate, and acceptable to use with frontline staff, such as medical assistants in specialty clinics, which was important to demonstrate, especially since we asked staff to adopt new professional roles for CVD risk reduction. Fidelity was evidenced by the facilitators’ documentation on copies of the IPAF tool, by staff’s responses to questionnaire items, and by the fact that staff’s perceived competence was higher after the implementations than prior to them. Adoption of target behaviors improved at 6 months and 24 months, including checking risk factors (i.e., BP and tobacco) and connecting patients to CVD risk reduction follow-up [13–16]. Such positive findings are consistent with the theories guiding our project and indicate that frontline staff such as medical assistants can be partners in implementing clinic protocols in specialty clinics and that our interactive and participatory A&F strategy can support such improvements.

### Limitations

Although this paper establishes strong multi-site implementation outcomes of a theory-based A&F strategy, we acknowledge limitations in our quasi-experimental design and the evaluation of IPAF in the context of other implementation components. Moreover, our reported sample size of staff receiving IPAF (*n* = 18) was modest. This limitation is offset by our multi-site findings representing eight implementations across two health systems, including 99 feedback sessions and more than 375 monthly feedback emails. Another limitation is that audit data for float staff were aggregated within staff data overall, yet float staff did not receive IPAF. Also, because of turnover, new staff received abridged engagement and education prior to IPAF, which may have diluted exposure to some SDT- and SRT-informed elements. Additionally, findings based on staff self-reports may be attributed to social desirability influences, given that the principal investigator was the IPAF facilitator in three of the four clinics. Consistent with Cochrane A&F recommendations for effective A&F [5], this facilitator was a respected colleague known to the staff within the three clinics. To reduce the risk of social desirability influence, the facilitator began each session anchoring the benefits of target behaviors for patient care and outcomes. In addition, the facilitator assured the staff that individual performance data remained confidential from supervisors. Similar staff target-behavior gains were confirmed with delivery by a nurse facilitator in a fourth clinic, suggesting efficacy and replicability beyond social desirability bias [14]. Finally, our pre- post-measurement of the staff’s perceived confidence was initially retrospective, which we addressed with prospective questionnaires in the fourth clinic.

### Future research

Researchers and clinical improvement teams can apply and extend our theory-based IPAF approach to evaluate improvements in staff adoption of other target behaviors in other settings. Teams can download our IPAF toolkit, including its tool and theoretical details, at https://www.hipxchange.org/AuditFeedback [35]. We plan a future clinic randomized controlled study, with active A&F comparisons of individual versus group feedback, and larger sample sizes, to further test these methods.

## Conclusions

We developed and evaluated IPAF, an interactive and participatory A&F strategy, to improve the implementation of evidence-based interventions, building on two complementary theories, SRT and SDT. Findings support feasibility, appropriateness, acceptability, and fidelity of IPAF, as well as staff adoption and maintenance of target behaviors. By evaluating implementation outcomes in multi-site specialty clinics with medical assistants and nurses performing pre-visit rooming, we have extended prior research on clinic protocols and A&F beyond primary care settings and providers. Our explication of how and why we applied the concepts from two complementary theories to IPAF can provide a strong foundation for future efforts. Researchers and clinical improvement teams can use IPAF to help improve clinical outcomes such as reducing CVD risk factors.

Reporting checklists: The STaRI and STROBE Checklists were used for reporting. (see Additional files).

## Supplementary Information

**Additional file 1.**

**Additional file 2.**

## Data Availability

The datasets used and/or analyzed during the current study are available from the corresponding author on reasonable request per IRB approval.

## Abbreviations

A&F: Audit and feedback
CVD: Cardiovascular disease
BP: Blood pressure
IPAF: Interactive and Participatory Audit and Feedback
EHR: Electronic health records
SRT: Self-regulation theory
SDT: Self-determination theory
TDF: Theoretical Domains Framework
